# Surviving the Heat of the Moment: A Fungal Pathogens Perspective

**DOI:** 10.1371/journal.ppat.1003163

**Published:** 2013-03-07

**Authors:** Michelle D. Leach, Leah E. Cowen

**Affiliations:** 1 Aberdeen Fungal Group, School of Medical Sciences, Institute of Medical Sciences, University of Aberdeen, Foresterhill, Aberdeen, United Kingdom; 2 Department of Molecular Genetics, University of Toronto, Toronto, Ontario, Canada; Duke University Medical Center, United States of America

## Introduction

Temperature is a critical parameter continually monitored by microorganisms. The dynamic environments inhabited by microorganisms evoke constant and effective environmental response strategies that have been elaborated over evolutionary time. For example, a significant rise or fall in ambient temperature initiates a stress response in the organism, commonly known as heat-shock or cold-shock responses, respectively. The phenomenon of temperature sensing has long been studied in microorganisms such as bacteria [1], but these mechanisms are only recently being translated to pathogenic fungi.

Fungal pathogens inhabit a remarkable diversity of environments and exhibit a dazzling repertoire of life cycles and environmentally contingent cellular processes. Take for example *Cryptococcus neoformans, Histoplasma capsulatum,* and *Aspergillus fumigatus*. These fungi are found in diverse environments such as pigeon excreta and soil, but retain a common denominator: the ability to grow at 37°C. Loss of genes necessary for high-temperature growth in these pathogens results in attenuated virulence and at times even death [2]–[4]. Therefore, high-temperature growth is essential for pathogenesis. A classic example is that *Saccharomyces cerevisiae* clinical isolates are able to grow at higher temperatures (41°C) than laboratory strains, a characteristic important for their survival in mice [5]. Besides governing virulence, temperature regulates many distinct processes. Morphological transitions and growth temperature are linked in the dimorphic fungi, such as *H. capsulatum*, which grow as filamentous molds at ambient temperature and switch to a yeast form at elevated host temperature [6]. Temperature also controls morphological transitions in the leading fungal pathogen of humans, *Candida albicans*; though for this organism ambient temperature favors the yeast form, while elevated temperature induces filamentous growth [7]. *C. albicans* can also switch from a white to opaque cellular state in host niches with lower temperatures, such as skin, facilitating mating [8]. Many genes are simply induced in response to elevated temperatures and do not serve to sense the external stimuli. Generally the consequences of a thermal upshift, as opposed to the temperature itself, provide a signal the cell will react to. This article focuses on how fungi sense and adapt to changing environmental temperature, using *S. cerevisiae* as a model.

## The Unfolded-Protein Response

**Figure 1:**
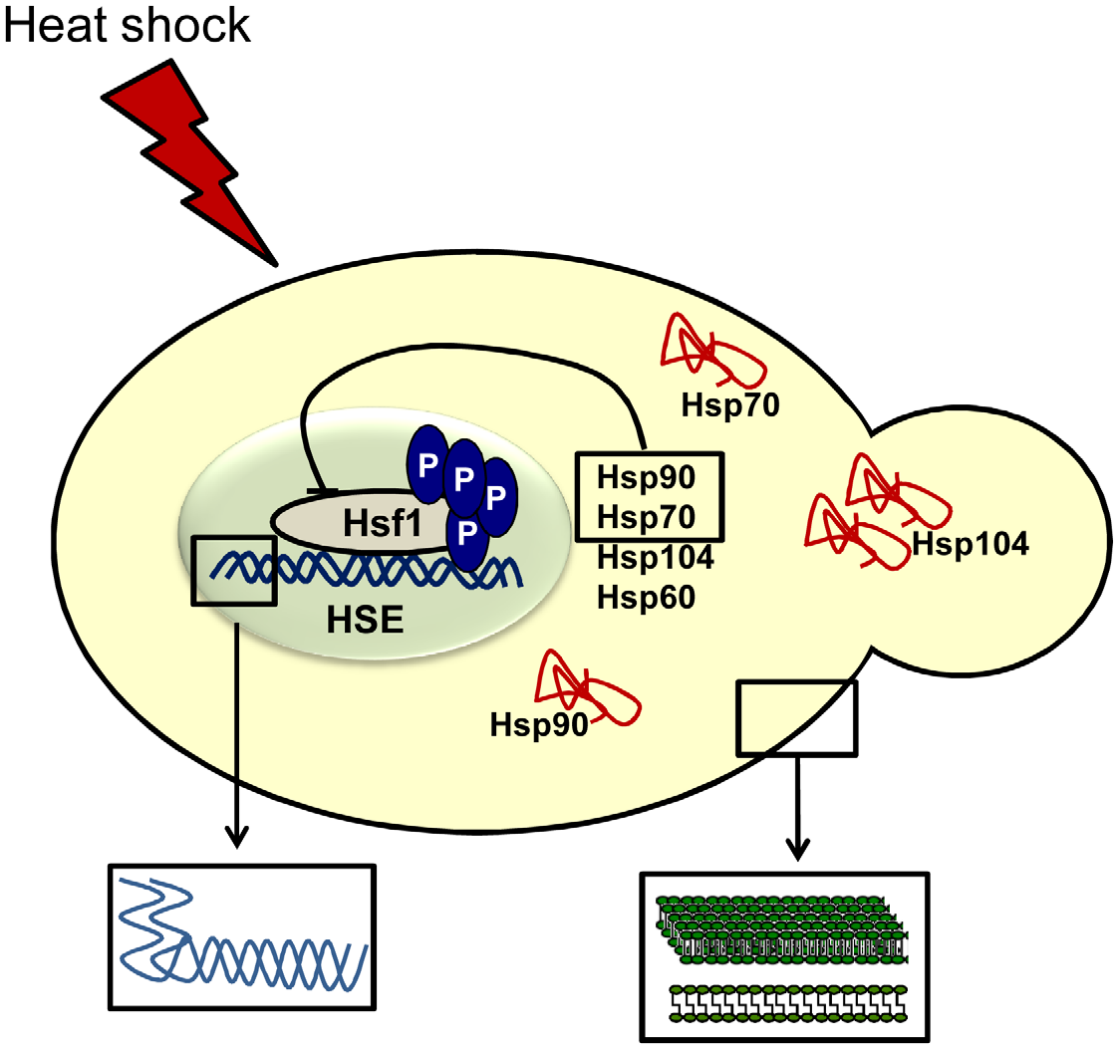
Mechanisms of temperature sensing in fungi. Upon a thermal insult, cells utilize different mechanisms to sense changes in the surrounding temperature for adaptation and survival. Hsf1 becomes phosphorylated, binding to heat-shock elements (HSEs) in target promoters, leading to the upregulation of chaperone proteins. These aid in the stabilization and refolding of denatured proteins, and Hsp90 and Hsp70 assist in the downregulation of Hsf1 [25]. The cellular plasma membrane becomes more fluid in response to thermal insults (right inset box). Moreover, although little is known about RNA thermometers in fungi, there is evidence suggesting they are involved in RNA decay during heat shock (left inset box) [20].

The heat-shock response has been universally conserved from bacteria to humans. Underpinning this phenomenon is that an increase in temperature contributes to protein denaturation in vivo. All living organisms respond by inducing a set of proteins important for high-temperature growth called heat-shock proteins, which, in eukaryotes, are dependent on the heat-shock transcription factor (Hsf1) (Figure 1) [9]–[11]. However, 25 years after the discovery of Hsf1 in *S. cerevisiae*, the specific trigger responsible for its activation has yet to be determined. Two recent studies on the unfolded-protein response in *S. cerevisiae* suggest that it is the location of unfolded proteins that determines activation of the heat-shock response. Using five transcriptional markers, Metzger and colleagues demonstrate that three different cellular compartments elicit distinct transcriptional responses upon protein denaturation [12]. Unfolded proteins within the endoplasmic reticulum (ER) of cells induce expression of *KAR2* as well as *HAC1* splicing, both early hallmarks of ER stress. In contrast, unfolded proteins within the cytosol lead to rapid upregulation of *SSA4*, the main Hsp70 chaperone in the cytosol, as well as *STI1*, a cytosolic cochaperone. This response is equivalent to that induced by heat shock [12]. The third response occurs when membrane proteins unfold. This does not lead to upregulation of any of the genes mentioned above, but instead induces a distinct class of genes. Following this study, Geiler-Samerotte and colleagues examined the proteomic response during chronic low-level expression of a misfolded YFP protein in *S. cerevisiae* [13]. They discovered that the majority of significantly induced proteins were cytosolic chaperones and cochaperones, including members of the Hsp70 family as well as Sti1. Indeed, 20 differentially regulated proteins identified in this study upon expression of a misfolded YFP are bound by Hsf1 [13]. Unfolded proteins are thus able to activate the classic heat-shock response, but only when found in the cytosol. This suggests that the cell may use multiple distinct mechanisms to sense temperature.

## Membrane Fluidity Dynamics

An alternative theory to unfolded proteins being the primary sensor of heat is changes in membrane structure and function. An abrupt increase in temperature triggers a rapid decrease in the molecular order of cellular membranes (i.e., fluidity increases). Studies on the fungal pathogen *H. capsulatum* found that exposure to saturated fatty acids alongside a rise in temperature leads to an increase in heat-shock gene transcription; conversely, addition of unsaturated fatty acids reduced heat-shock gene transcription during a temperature upshift [14]. These authors also complemented a *S. cerevisiae ole1/ole1* mutant that lacks a fatty acid desaturase using constructs with the native promoter or two *H. capsulatum* promoters, which express *OLE1* at different levels [15]. Complemented strains modified the saturated versus unsaturated fatty acids ratio, changing the physical state of cellular membranes and demonstrating a different threshold temperature of heat-shock gene expression relative to the mutant [14]. Although no such work has been done in *C. albicans* or *C. neoformans*, transcriptional-profiling studies of *C. neoformans* during high-temperature growth identified the transcriptional activator *MGA2* [16]. In *S. cerevisiae*, Mga2 and its homologue Spt23 regulate transcription of *OLE1* [17], suggesting that *C. neoformans* may require cell-membrane remodelling to mitigate responses to temperature change. However, the time scale over which changes in membrane fluidity occur may not be rapid enough for this to serve as primary sensor of temperature.

## RNA Thermometers

An increase in temperature to 37°C signals to pathogenic microorganisms that they have successfully invaded a warm-blooded mammalian host, leading to expression of virulence genes. In bacteria, RNA structures are a key element of the heat-shock response. They can occlude RNA binding sites in mRNAs of key regulators that melt during heat shock to enable protein synthesis [18]. These have been termed RNA thermometers. Indeed, the three major classes of temperature-responsive genes, including heat-shock genes, cold-shock genes, and genes necessary for virulence, control temperature-dependent translation initiation by exploiting RNA thermometers [19]. Until very recently the number and location of RNA thermometers in eukaryotes remained unknown. However, a study by Wan and colleagues identified thousands of putative RNA thermometers by probing *S. cerevisiae* RNA structures at different temperatures [20]. In keeping with transcript-profiling data, genes with low melting temperatures were enriched for ribosomal proteins, whereas transcripts with higher melting temperatures included key unfolded-protein response activators such as *HAC1* [20]. Strikingly, Wan and colleagues showed that the most structurally stable mRNAs barely decline during heat shock, whereas mRNAs with low melting temperatures displayed considerable and prolonged decreases in transcript levels. They concluded that RNA thermometers in *S. cerevisiae* play a role in RNA decay during heat shock [20]. However, whether these mechanisms contribute to temperature sensing in fungal pathogens remains to be determined. Despite the central importance of RNA thermometers for temperature-dependent gene regulation, it is clear that there are other key mechanisms as it is unlikely that RNA thermometers account for activation of Hsf1, the transcription factor that commands the heat-shock response.

## Hsf1-Hsp90 Autoregulatory Circuit

The heat-shock response is governed by Hsf1, an essential protein in yeast, which is activated by phosphorylation upon a thermal upshift (Figure 1) [10]. In *C. albicans*, Hsf1 is phosphorylated rapidly (within one minute) of a 30–42°C heat shock. It is a relatively transient response, downregulated upon cellular adaptation [21]. The means of this downregulation have followed a model whereby two classes of heat-shock proteins have been implicated in regulation of Hsf1 activity. The first class is Hsp70. In *S. cerevisiae*, deletion of the two cytosolic Hsp70 isoforms *SSA1* and *SSA2* induces Hsf1 transcriptional activity [22]. Hsp70 operates within the Hsp90 chaperone machine, which stabilizes a wide range of cellular clients [23]. Mutations that interfere with Hsp90 function in *S. cerevisiae* cause increased levels of Hsf1 activity [24]. However, it is frequently argued that interfering with major cytosolic chaperone proteins generates an unfolded-protein response, which could be responsible for de-repressing Hsf1 transcriptional activity. Until recently no physical interaction between Hsf1 and either Hsp90 or Hsp70 had been observed in yeast. Leach and colleagues utilized the fungal pathogen *C. albicans* to determine genetically and biochemically that Hsf1 interacts with Hsp90 under steady-state conditions, and that upon thermal insults, this interaction is strengthened [25]. This stands in contrast to the model that Hsf1 activation requires release from Hsp90, but is consistent with a recent perspective on the impact of temperature on the Hsp90 interactome [26]. Leach and colleagues also detected an interaction between Hsp90, Hsf1, and Hsp70 when cells were exposed to a 60- or 120-minute heat shock at 42°C, and that Hsp90 localised to the nucleus during elevated temperatures. Together, this provides the strongest evidence to date for the existence of the Hsf1-Hsp90 autoregulatory circuit. However, as Hsf1 did not dissociate from Hsp90 upon heat shock, it is unlikely that Hsp90 directly controls the rapid activation of Hsf1. Indeed, it is feasible that the kinase responsible for activating Hsf1 serves as the immediate sensor of temperature, yet the identity of this kinase has remained elusive since the discovery that Hsf1 is activated by phosphorylation.

## Beyond Temperature Sensing

There have been many advances in understanding temperature regulation in eukaryotes over the years, although numerous enigmas remain to be resolved. Despite the high degree of conservation of many components of the heat-shock response, the biology of temperature regulation in fungi is remarkably distinct from mammalian systems. Although studies in *S. cerevisiae* have provided a foundation for understanding how cells sense temperature shifts, whether this regulation is conserved in medically relevant fungi needs to be addressed given the importance of high temperatures for infection. The most fundamental challenge is to understand how the cell senses temperature and initiates the appropriate cellular response. For example, does an increase in membrane fluidity act as the primary sensor of temperature, or is it a response for adaptation? How rapidly do these changes occur? Which kinase phosphorylates Hsf1? The rapid timescale of Hsf1 phosphorylation suggests the possibility of a temperature-sensing kinase, whose activity might be exquisitely sensitive to temperature-dependent changes in structure. Understanding the mechanisms of temperature sensing in pathogens remains a priority and could lead to novel drug targets.

## References

[1] Klinkert B, Narberhaus F (2009) Microbial thermosensors. Cell Mol Life Sci 66: 2661–2676. 19554260 10.1007/s00018-009-0041-3 PMC11115684

[2] Bhabhra R, Miley MD, Mylonakis E, Boettner D, Fortwendel J, et al (2004) Disruption of the *Aspergillus fumigatus* gene encoding nucleolar protein CgrA impairs thermotolerant growth and reduces virulence. Infect Immun 72: 4731–4740. 15271935 10.1128/IAI.72.8.4731-4740.2004 PMC470587

[3] Lamoth F, Juvvadi PR, Fortwendel JR, Steinbach WJ (2012) Heat shock protein 90 is required for conidiation and cell wall integrity in *Aspergillus fumigatus* . Eukaryot Cell 11: 1324–1332. 22822234 10.1128/EC.00032-12 PMC3486032

[4] Odom A, Muir S, Lim E, Toffaletti DL, Perfect J, et al (1997) Calcineurin is required for virulence of *Cryptococcus neoformans* . EMBO J 16: 2576–2589. 9184205 10.1093/emboj/16.10.2576 PMC1169869

[5] McCusker JH, Clemons KV, Stevens DA, Davis RW (1994) *Saccharomyces cerevisiae* virulence phenotype as determined with CD-1 mice is associated with the ability to grow at 42°C and form pseudohyphae. Infect Immun 62: 5447–5455. 7960125 10.1128/iai.62.12.5447-5455.1994 PMC303287

[6] Maresca B, Kobayashi GS (1989) Dimorphism in *Histoplasma capsulatum*: A model for the study of cell differentiation in pathogenic fungi. Microbiol Rev 53: 186–209. 2666842 10.1128/mr.53.2.186-209.1989 PMC372727

[7] Gow NA, Brown AJ, Odds FC (2002) Fungal morphogenesis and host invasion. Curr Opin Microbiol 5: 366–371. 12160854 10.1016/s1369-5274(02)00338-7

[8] Lachke SA, Lockhart SR, Daniels KJ, Soll DR (2003) Skin facilitates *Candida albicans* mating. Infect Immun 71: 4970–4976. 12933839 10.1128/IAI.71.9.4970-4976.2003 PMC187354

[9] Wu C (1995) Heat shock transcription factors: Structure and regulation. Annu Rev Cell Dev Biol 11: 441–469. 8689565 10.1146/annurev.cb.11.110195.002301

[10] Sorger PK, Pelham HRB (1988) Yeast heat shock factor is an essential DNA-binding protein that exhibits temperature-dependent phosphorylation. Cell 54: 855–864. 3044613 10.1016/s0092-8674(88)91219-6

[11] Nicholls S, Leach MD, Priest CL, Brown AJ (2009) Role of the heat shock transcription factor, Hsf1, in a major fungal pathogen that is obligately associated with warm-blooded animals. Mol Microbiol 74: 844–861. 19818013 10.1111/j.1365-2958.2009.06883.x PMC3675641

[12] Metzger MB, Michaelis S (2009) Analysis of quality control substrates in distinct cellular compartments reveals a unique role for Rpn4p in tolerating misfolded membrane proteins. Mol Biol Cell 20: 1006–1019. 19073890 10.1091/mbc.E08-02-0140 PMC2633399

[13] Geiler-Samerotte KA, Dion MF, Budnik BA, Wang SM, Hartl DL, et al (2011) Misfolded proteins impose a dosage-dependent fitness cost and trigger a cytosolic unfolded protein response in yeast. Proc Natl Acad Sci U S A 108: 680–685. 21187411 10.1073/pnas.1017570108 PMC3021021

[14] Carratu L, Franceschelli S, Pardini CL, Kobayashi GS, Horvath I, et al (1996) Membrane lipid perturbation modifies the set point of the temperature of heat shock response in yeast. Proc Natl Acad Sci U S A 93: 3870–3875. 8632982 10.1073/pnas.93.9.3870 PMC39451

[15] Gargano S, Di Lallo G, Kobayashi GS, Maresca B (1995) A temperature-sensitive strain of *Histoplasma capsulatum* has an altered Δ9-fatty acid desaturase gene. Lipids 30: 899–906. 8538376 10.1007/BF02537480

[16] Kraus PR, Boily MJ, Giles SS, Stajich JE, Allen A, et al (2004) Identification of *Cryptococcus neoformans* temperature-regulated genes with a genomic-DNA microarray. Eukaryot Cell 3: 1249–1260. 15470254 10.1128/EC.3.5.1249-1260.2004 PMC522612

[17] Zhang S, Skalsky Y, Garfinkel DJ (1999) *MGA2* or *SPT23* is required for transcription of the Δ9 fatty acid desaturase gene, *OLE1*, and nuclear membrane integrity in *Saccharomyces cerevisiae* . Genetics 151: 473–483. 9927444 10.1093/genetics/151.2.473 PMC1460504

[18] Chowdhury S, Maris C, Allain FH, Narberhaus F (2006) Molecular basis for temperature sensing by an RNA thermometer. EMBO J 25: 2487–2497. 16710302 10.1038/sj.emboj.7601128 PMC1478195

[19] Kortmann J, Narberhaus F (2012) Bacterial RNA thermometers: molecular zippers and switches. Nat Rev Microbiol 10: 255–265. 22421878 10.1038/nrmicro2730

[20] Wan Y, Qu K, Ouyang Z, Kertesz M, Li J, et al (2012) Genome-wide measurement of RNA folding energies. Mol Cell 48: 169–181. 22981864 10.1016/j.molcel.2012.08.008 PMC3483374

[21] Leach MD, Tyc KM, Brown AJP, Klipp E (2012) Modelling the regulation of thermal adaptation in *Candida albicans*, a major fungal pathogen of humans. PLoS ONE 7: e32467 doi:10.1371/journal.pone.0032467. 22448221 10.1371/journal.pone.0032467 PMC3308945

[22] Craig EA, Jacobsen K (1984) Mutations of the heat inducible 70 kilodalton genes of yeast confer temperature sensitive growth. Cell 38: 841–849. 6386178 10.1016/0092-8674(84)90279-4

[23] Taipale M, Krykbaeva I, Koeva M, Kayatekin C, Westover KD, et al (2012) Quantitative analysis of Hsp90-client interactions reveals principles of substrate recognition. Cell 150: 987–1001. 22939624 10.1016/j.cell.2012.06.047 PMC3894786

[24] Duina AA, Kalton HM, Gaber RF (1998) Requirement for Hsp90 and a CyP-40-type cyclophilin in negative regulation of the heat shock response. J Biol Chem 273: 18974–18978. 9668076 10.1074/jbc.273.30.18974

[25] Leach MD, Budge S, Walker L, Munro C, Cowen LE, et al (2012) Hsp90 orchestrates transcriptional regulation by Hsf1 and cell wall remodelling by MAPK signalling during thermal adaptation in a pathogenic yeast. PLoS Pathog 8: e1003069 doi:10.1371/journal.ppat.1003069. 23300438 10.1371/journal.ppat.1003069 PMC3531498

[26] Leach MD, Klipp E, Cowen LE, Brown AJ (2012) Fungal Hsp90: A biological transistor that tunes cellular outputs to thermal inputs. Nat Rev Microbiol 10: 693–704. 22976491 10.1038/nrmicro2875 PMC3660702

